# Ultrasensitive detection of salivary SARS-CoV-2 IgG antibodies in individuals with natural and COVID-19 vaccine-induced immunity

**DOI:** 10.1038/s41598-022-12869-z

**Published:** 2022-05-25

**Authors:** Stefani N. Thomas, Amy B. Karger, Ghaith Altawallbeh, Kathryn M. Nelson, David R. Jacobs, Jed Gorlin, Helene Barcelo, Bharat Thyagarajan

**Affiliations:** 1 Department of Laboratory Medicine and Pathology, School of Medicine, University of Minnesota, 420 Delaware St. SE MMC 609, Minneapolis, MN 55455 USA; 2 Institute for Therapeutics Discovery and Development, College of Pharmacy, University of Minnesota, Minneapolis, MN USA; 3 Division of Epidemiology and Community Health, School of Public Health, University of Minnesota, Minneapolis, MN USA; 4 Memorial Blood Centers—A Division of New York Blood Center Enterprises, St. Paul, MN USA; 5 Present Address: Intermountain Central Laboratory, Murray, UT USA

**Keywords:** Biochemical assays, Proteins

## Abstract

We assessed the feasibility of a highly sensitive immunoassay method based on single molecule array (Simoa) technology to detect IgG and IgA antibodies against SARS-CoV-2 spike protein receptor binding domain (RBD) in saliva from individuals with natural or vaccine-induced COVID-19 immunity. The performance of the method was compared to a laboratory-developed SARS-CoV-2 RBD total antibody enzyme-linked immunosorbent assay (ELISA). Paired serum and saliva specimens were collected from individuals (n = 40) prior to and 2 weeks after receiving an initial prime COVID-19 vaccine dose (Pfizer/BioNTech BNT162b2 or Moderna mRNA-1273). Saliva was collected using a commercially available collection device (OraSure Inc.) and SARS-CoV-2 RBD IgG antibodies were measured by an indirect ELISA using concentrated saliva samples and a Simoa immunoassay using unconcentrated saliva samples. The IgG results were compared with paired serum specimens that were analyzed for total RBD antibodies using the ELISA method. The analytical sensitivity of the saliva-based Simoa immunoassay was five orders of magnitude higher than the ELISA assay: 0.24 pg/mL compared to 15 ng/mL. The diagnostic sensitivity of the saliva ELISA method was 90% (95% CI 76.3–97.2%) compared to 91.7% (95% CI 77.5–98.2%) for the Simoa immunoassay without total IgG-normalization and 100% (95% CI 90.3–100%) for the Simoa immunoassay after total IgG-normalization when compared to the serum ELISA assay. When analyzed using the SARS-CoV-2 RBD IgG antibody ELISA, the average relative increase in antibody index (AI) between the saliva of the post- and pre-vaccinated individuals was 8.7 (AI_post/pre_). An average relative increase of 431 pg/mL was observed when the unconcentrated saliva specimens were analyzed using the Simoa immunoassay (SARS-CoV-2 RBD IgG_post/pre_). These findings support the suitability of concentrated saliva specimens for the measurement of SARS-CoV-2 RBD IgG antibodies via ELISA, and unconcentrated saliva specimens for the measurement of SARS-CoV-2 RBD IgG and IgA using an ultrasensitive Simoa immunoassay.

## Introduction

The COVID-19 pandemic is unprecedented and continues to be a global public health emergency that has affected more than 230 million people worldwide, resulting in 4.7 million deaths as of September 2021^1–3^. Although serological assays are not intended for diagnostic purposes, antibody detection can be useful to evaluate the degree of immunization, measure seroprevalence, identify and trace contacts, and identify potential convalescent plasma donors^4,5^. The majority of serological tests for antibodies against SARS-CoV-2 require a blood sample from an invasive venipuncture, which cannot be readily implemented in large population-based screening programs.

Saliva is a clear mixture of extracellular secretions produced by salivary glands in the mouth that consists of approximately 99% water, while the remaining components consist of electrolytes, mucus, and protein including antimicrobial agents such as IgA and IgG^6–8^. It is a noninvasive alternative sample source that offers the flexibility of self-collection and home-based collection. There is a precedent for the utility of saliva for the detection of antibodies against various viral agents^9–11^. For example, good correlation has been reported between salivary and serum antibodies for human immunodeficiency virus (HIV) antibody testing^9,12–14^. Recent studies have shown a strong correlation between antibodies against SARS-CoV-2 in blood and saliva^15–18^.

With the increased deployment of vaccines against SARS-CoV-2, there is interest in determining vaccine efficacy and duration of immunity-related protection. Given that the currently available vaccines generate an immune response to SARS-CoV-2 spike antigens, anti-spike IgG antibody levels, which have been demonstrated to correlate with neutralizing activity, provide a potential surrogate marker of protection^19–21^. Data show that individuals vaccinated with the Pfizer/BioNTech BNT162b2 and Moderna mRNA-1273 vaccines seroconvert within 21 days of the prime dose, with anamnestic immune responses and higher antibody titers observed in individuals previously infected with COVID-19^22,23^. To facilitate the non-invasive evaluation of vaccine efficacy and duration of immunity-related protection, we validated a SARS-CoV-2 spike protein receptor binding domain (RBD) IgG antibody enzyme-linked immunosorbent assay (ELISA) method and a highly sensitive immunoassay method based on single molecule array (Simoa) technology to detect IgG antibodies against SARS-CoV-2 spike protein receptor binding domain (RBD) in saliva from individuals with natural or vaccine-induced COVID-19 immunity. Assay performance was evaluated by comparing the relative antibody levels in saliva to matched serum samples in 40 individuals prior to and 2 weeks after receiving an initial prime COVID-19 vaccine dose (Pfizer/BioNTech BNT162b2 or Moderna mRNA-1273) and 26 convalescent plasma donors who recovered from COVID-19 infection.

## Methods

### Study sample

Paired serum and saliva specimens were obtained from a cohort of 40 donors at the University of Minnesota prior to and 2 weeks after receiving an initial COVID-19 vaccine dose (Pfizer/BioNTech BNT162b2 or Moderna mRNA-1273) [Age range: 18–65; 40% male, 60% female]. Two of the donors had PCR-confirmed COVID-19 infection prior to vaccination. The other 38 donors reported no history of COVID-19 infection. All study participants gave informed consent. In addition, paired serum and saliva were obtained from the Memorial Blood Centers in St. Paul, MN from convalescent plasma donors (n = 26) who recovered from COVID-19 infection. The University of Minnesota Institutional Review Board (IRB) determined that this study was not considered human subjects research. Sample collections at Memorial Blood Centers were approved by their IRB.

### Saliva and blood sample collection

All methods described herein were performed in accordance with relevant guidelines and regulations. Saliva samples were collected with OraSure oral specimen collection devices (Catalog # 3001-0886, OraSure Technologies) according to the manufacturer’s instructions. In brief, saliva was collected with a fiber pad. The pad was brushed between the cheek and lower gum and held in place for 2 min. Then the pad was placed into a transport tube containing a preservative solution and processed within 24 h of collection. The collection devices were centrifuged at 3,100 rpm in a Heraeus Multifuge (Thermo Fisher Scientific) for 10 min, to obtain an eluted volume of ~ 1.6–1.8 mL that was transferred to a 2 mL cryovial for storage. To obtain detectable levels of SARS-CoV-2 RBD IgG antibodies in saliva using our laboratory-developed ELISA method, it was necessary to further concentrate the saliva samples. Concentration was achieved using an Ultra-4 Centrifugal Filter Device (Catalog # UFC805024, Millipore Sigma), 50 K NMWL at 4,000 RPM for 15 min to a final volume of 100 µL. Blood samples were collected into serum separator tubes (SST), and processed to yield serum according to standard clinical laboratory procedures. All samples were stored at -80 °C until use.

### Detection of SARS-CoV-2 antibodies in serum and saliva with a laboratory-developed ELISA method

All serum and saliva samples were measured using our laboratory-developed ELISA, the details of which are described elsewhere^24^. To date, this assay has been used to measure SARS-CoV-2 RBD total antibody levels in > 25,000 specimens in our laboratory. Of note, all serum samples were tested for total SARS-CoV-2 antibodies while saliva samples were tested only for IgG SARS-CoV-2 antibodies as total antibody measurements resulted in a high background and a substantial number of false positive results (data not shown). In brief, ELISA plates were coated with recombinant SARS-CoV-2 spike RBD protein as the antigen. Patient total RBD protein antibodies (serum) or IgG antibodies (saliva) were recognized by goat anti-human IgG H + L-HRP (Invitrogen, A18805). Antibodies extracted from saliva collection devices were analyzed alongside paired serum samples on the same ELISA plate. Prior to analysis of participant saliva samples, a dose–response study was conducted to assess assay linearity, limit of detection, and potential matrix effects in saliva using our laboratory-developed ELISA. For analysis of participant samples, 50 µL of concentrated saliva versus 1 µL of serum (diluted 1:50 fold) was used. The Antibody Index (AI) for all samples was calculated by dividing each sample’s OD_450nm_ by the mean of the pre-pandemic pooled serum control. For saliva samples, antibody indices ≥ 2.8 were categorized as positive and values < 2.8 were categorized as negative. The positivity cutoff value was defined as the mean + (3 × standard deviation) of the AI of the true negative samples.

### Measurement of salivary total IgG

A commercially available salivary total IgG ELISA kit was used for the quantitative measurement of human total IgG (Salimetrics Inc.), to normalize SARS-CoV-2 salivary IgG antibody levels to total IgG. The indirect sandwich ELISA kit was used according to the manufacturer’s instructions. Briefly, pre-coated capture anti-human IgG antibody present on the plate was used to bind IgG in samples, which was then bound by an anti-human IgG detection antibody linked to horseradish peroxidase. Following washing after each incubation step, bound anti-human IgG antibody enzyme conjugate was added and the levels measured by the reaction of the horseradish peroxidase enzyme with the substrate, tetramethylbenzidine (TMB). The OD_450nm_ was read on a standard plate reader. The amount of IgG antibody enzyme conjugate detected was directly proportional to the amount of total human IgG present in the sample.

### Simoa SARS-CoV-2 spike RBD protein IgG and IgA antibody immunoassay

To evaluate another analytical method with higher sensitivity than the laboratory-developed ELISA for the measurement of SARS-CoV-2 RBD IgG antibodies, we developed a single molecule digital ELISA Simoa assay. Analytes have been detected in serum at sub-femtomolar concentrations using Simoa methods^25^. A Simoa 3-step digital immunoassay was utilized for the quantitative determination of SARS-CoV-2 spike RBD protein IgG and IgA antibodies in saliva using a Quanterix HD-X analyzer. The saliva samples used for this assay were neither concentrated nor diluted. The paramagnetic beads, diluent, bead conjugation and wash buffer, biotinylation reaction buffer, streptavidin-ß-galactosidase (SBG), resorufin-ß-D-galactopyranoside (RGP), 1-ethyl-3-(3-dimethylaminopropyl) carbodiimide hydrochloride (EDC), and EZ-Link biotin were provided in a Simoa homebrew assay starter kit (Quanterix). Briefly, carboxylated paramagnetic beads were coated with SARS-CoV-2 RBD protein (GenScript). SARS-CoV-2 IgG spike antibody CR3022 (Novus Biologicals) for IgG detection or SARS-CoV-2 IgA spike antibody CR3022 (Invivogen) for IgA detection was used as the calibrator and biotin-conjugated goat-anti-human IgG or IgA (Invitrogen) was used as the detection antibody. SBG was used for enzyme labeling of the captured target. The beads were re-suspended in an RGP substrate solution prior to transferring to a Simoa Disc where the beads were sealed within microwells in the array. The concentration of SARS-CoV-2 RBD IgG or IgA antibody in the samples was interpolated from a calibration curve based on the average enzyme per bead (AEB).

### Data analysis

Assay diagnostic performance for salivary assays was determined based on sensitivity [(true positives)/(true positives + false negatives)], specificity [(true negatives)/(true negatives + false positives)], negative predictive value [(true negatives)/(true negatives + false negatives)] and positive predictive value [(true positives)/(true positives + false positives)], using the serum assay as the gold standard. The serum assay was selected as the gold standard for our study based on prior data demonstrating overall superior assay performance of serum antibody detection assays relative to salivary antibody detection assays in individuals with PCR-confirmed SARS-CoV-2 infection^17^. Therefore, test results from specimens that were positive for SARS-CoV-2 antibodies in saliva but negative in serum were categorized as false positives. Conversely, test results from specimens that were positive in serum but negative in saliva were categorized as false negatives. True positive saliva test results were defined as test results that were also positive in matched serum samples using a previously validated home-brew ELISA assay, whereas true negative saliva test results were defined as those that were negative in matched serum samples using a validated home-brew ELISA assay. Statistical analyses were conducted using the Data Analysis ToolPak in Excel. The statistical significance of *p*-values was assessed at an alpha of 0.05.

## Results

### Linearity studies and limit of detection in saliva using the laboratory-developed SARS-CoV-2 RBD total antibody ELISA

A dose–response relationship was observed between CR3022 concentrations in saliva and optical density (OD) at 450 nm similar to our validated serum assay^24^; therefore, no evidence of matrix effect was observed. There was a linear response in the range of 0.01–0.25 µg/mL, while at higher concentrations of CR3022, the response plateaued indicating signal saturation. The lower limit of detection (LLOD) of the assay was determined to be 15 ng/mL.

### Diagnostic performance of salivary SARS-CoV-2 RBD antibody ELISA

#### Pre- and post-COVID-19 vaccination samples

To determine whether anti-SARS-CoV-2 antibody responses could be detected in the saliva of individuals with vaccine-induced COVID-19 immunity, we compared the relative antibody levels in saliva to matched serum samples in 40 individuals prior to and 2 weeks after receiving an initial prime COVID-19 vaccine dose (Pfizer/BioNTech BNT162b2 or Moderna mRNA-1273). Among this group of individuals, two had PCR-confirmed COVID-19 infection prior to vaccination; both the saliva and matched serum samples from these two individuals tested positive prior to vaccination. The remaining participants had no known prior history of COVID-19 infection.

**Figure 1:**
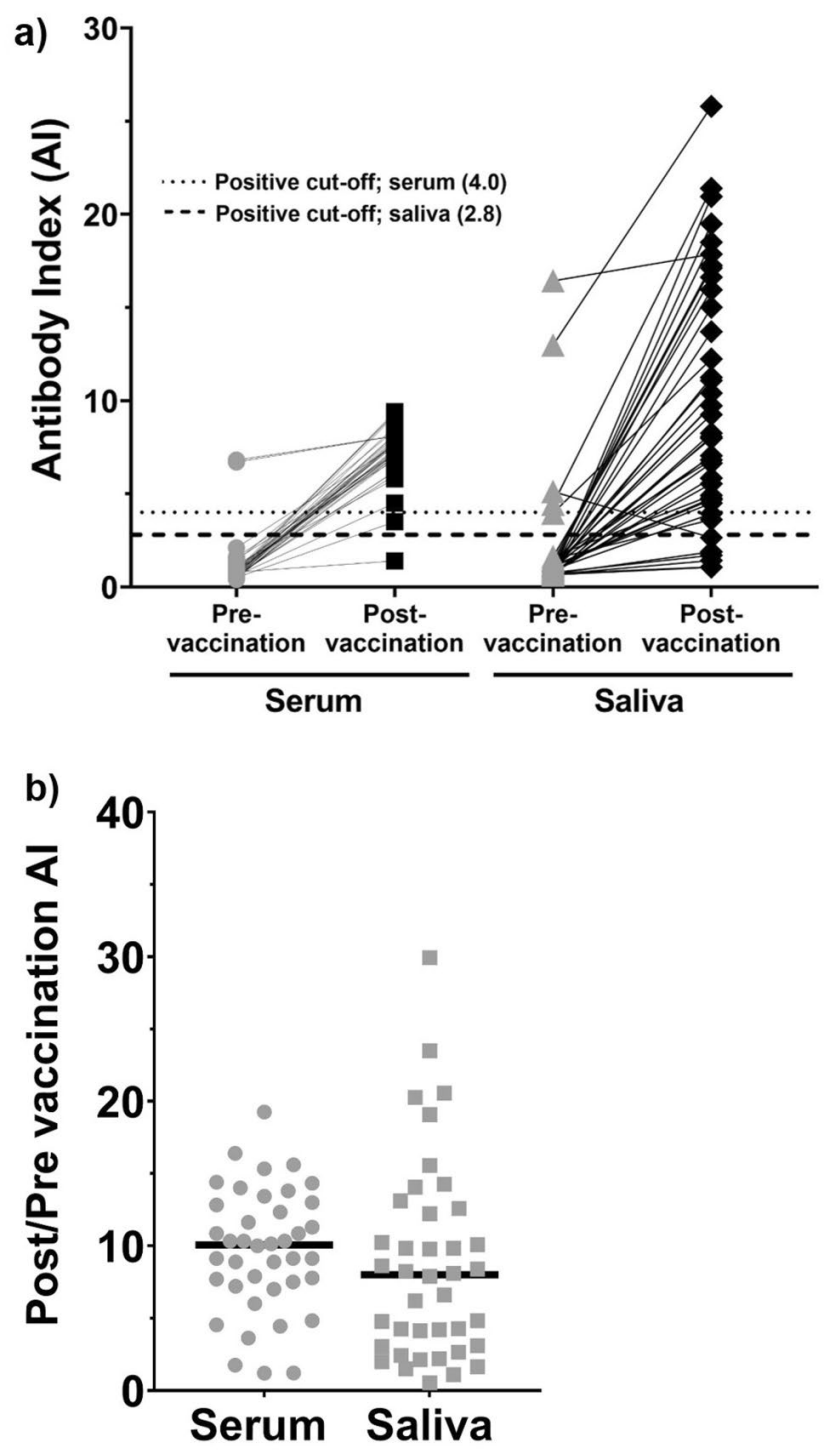
Distribution of SARS-CoV-2 spike protein RBD total antibody AI values using a home-brew ELISA in pre- and post-COVID-19 vaccination serum and saliva samples. Serum and saliva were obtained from 40 individuals prior to and after COVID-19 vaccination. Antibody indices were measured using a laboratory-developed SARS-CoV-2 total antibody ELISA. The positive antibody index cut-off for serum samples was 4.0 and 2.8 for saliva samples. (**a**) Changes in AI pre- and post-vaccination in serum and saliva in the context of the positive cut-off value for each sample type. (**b**) Pre- vs. post-vaccination AI ratios in serum and saliva.

Increased AIs were observed in the serum and saliva post-vaccination compared to pre-vaccination (Fig. 1a). The mean AI fold increase in the serum samples was 9.7 versus 8.7 in the saliva (Fig. 1b). The highest salivary AIs post-vaccination were observed in the saliva from the two individuals with previous natural COVID-19 immunity; however, this phenomenon was not observed in the serum. Eight of the 38 COVID-19 infection naïve individuals had higher post-vaccination serum AI values than the two individuals with confirmed COVID-19 infection prior to vaccination.

Performance characteristics of the saliva-based SARS-CoV-2 IgG antibody ELISA were calculated using the serum ELISA results as the gold standard. The diagnostic sensitivity of the saliva-based SARS-CoV-2 IgG antibody ELISA was 100% (95% CI 15.8–100%), 89.5% (95% CI 75.2–97.1%), and 90% (95% CI 76.3–97.2%) among the pre-vaccinated individuals, post-vaccinated individuals, and the overall cohort, respectively (Table 1). The diagnostic specificity among these groups was 92.1% (95% CI 78.6–98.3%), 100% (95% CI 15.8–100%), and 92.5% (95% CI 79.6–98.4%), respectively.

**Table 1:** Sensitivity and specificity diagnostic performance characteristics of the saliva-based IgG home-brew ELISA, IgG Simoa assay without IgG normalization, IgG Simoa assay with IgG normalization and IgA Simoa assay.

	Pre-vaccination		Post-vaccination		Overall	
	Sensitivity	Specificity	Sensitivity	Specificity	Sensitivity	Specificity
IgG Home-brew ELISA	100% [15.8–100%] (2/2 + 0)	92.1% [78.6–98.3%] (35/35 + 3)	89.5% [75.2–97.1%] (34/34 + 4)	100% [15.8–100%] (2/2 + 0)	90% [76.3–97.2%] (36/36 + 4)	92.5% [79.6–98.4%] (37/37 + 3)
IgG Simoa assay (without IgG normalization)	100% [15.8 –100%] (2/2 + 0)	97.1% [84.7–99.9%] (33/33 + 1)	91.2% [76.3–98.1%] (31/31 + 3)	NA (0/0 + 0)	91.7% [77.5–98.2%] (33/33 + 3)	97.1% [84.7–99.9%] (33/33 + 1)
IgG Simoa assay (with IgG normalization)	100% [15.8 –100%] (2/2 + 0)	94.1% [80.3–99.3%] (32/32 + 2)	100% [89.7–100.0%] (34/34 + 0)	NA (0/0 + 0)	100% [90.3–100%] (36/36 + 0)	94.1% [80.3–99.3%] (32/32 + 2)
IgA Simoa assay	50.0% [1.26–98.74%] (1/1 + 1)	96.7% [84.2–99.9%] (32/32 + 1)	41.2% [24.6–59.3%] (14/14 + 20)	NA (0/0 + 0)	41.7% [25.5—59.2%] (15/15 + 21)	97.0% [84.2–99.9%] (32/32 + 1)

Sensitivity = (True positives/True positives + False negatives). Specificity = (True negatives/True negatives + False positives). [95% confidence interval].

*IgG Home-brew assay:* Two individuals had PCR-confirmed COVID-19 infection prior to vaccination. Both of these saliva samples tested positive in serum and saliva. All other participants (n = 38) were confirmed to be COVID-19 negative based on PCR testing. Antibody-positive saliva samples from individuals with antibody-negative serum were considered to be false-positive results. Saliva samples were concentrated prior to analysis.

*IgG Simoa assay without IgG normalization:* The levels of SARS-CoV-2 RBD IgG were measured in paired serum and saliva samples from 36 pre-vaccinated individuals, serum from 35 post-vaccinated individuals, and saliva from 34 post-vaccinated individuals. Saliva samples were not concentrated prior to analysis.

*IgG Simoa assay with IgG normalization:* The levels of SARS-CoV-2 RBD IgG were measured in paired serum and saliva samples from 36 pre-vaccinated individuals, serum from 35 post-vaccinated individuals, and saliva from 34 post-vaccinated individuals (paired serum and saliva samples were available from 33 individuals). Saliva samples were not concentrated prior to analysis. Saliva SARS-CoV-2 spike RBD protein IgG levels were normalized to total IgG levels.

*IgA Simoa assay:* The levels of SARS-CoV-2 RBD IgA were measured in paired serum and saliva samples from 35 pre-vaccinated individuals, and 34 post-vaccinated individuals. Saliva samples were not concentrated prior to analysis.

#### Saliva samples from individuals who recovered from natural COVID-19 infection

**Figure 2:**
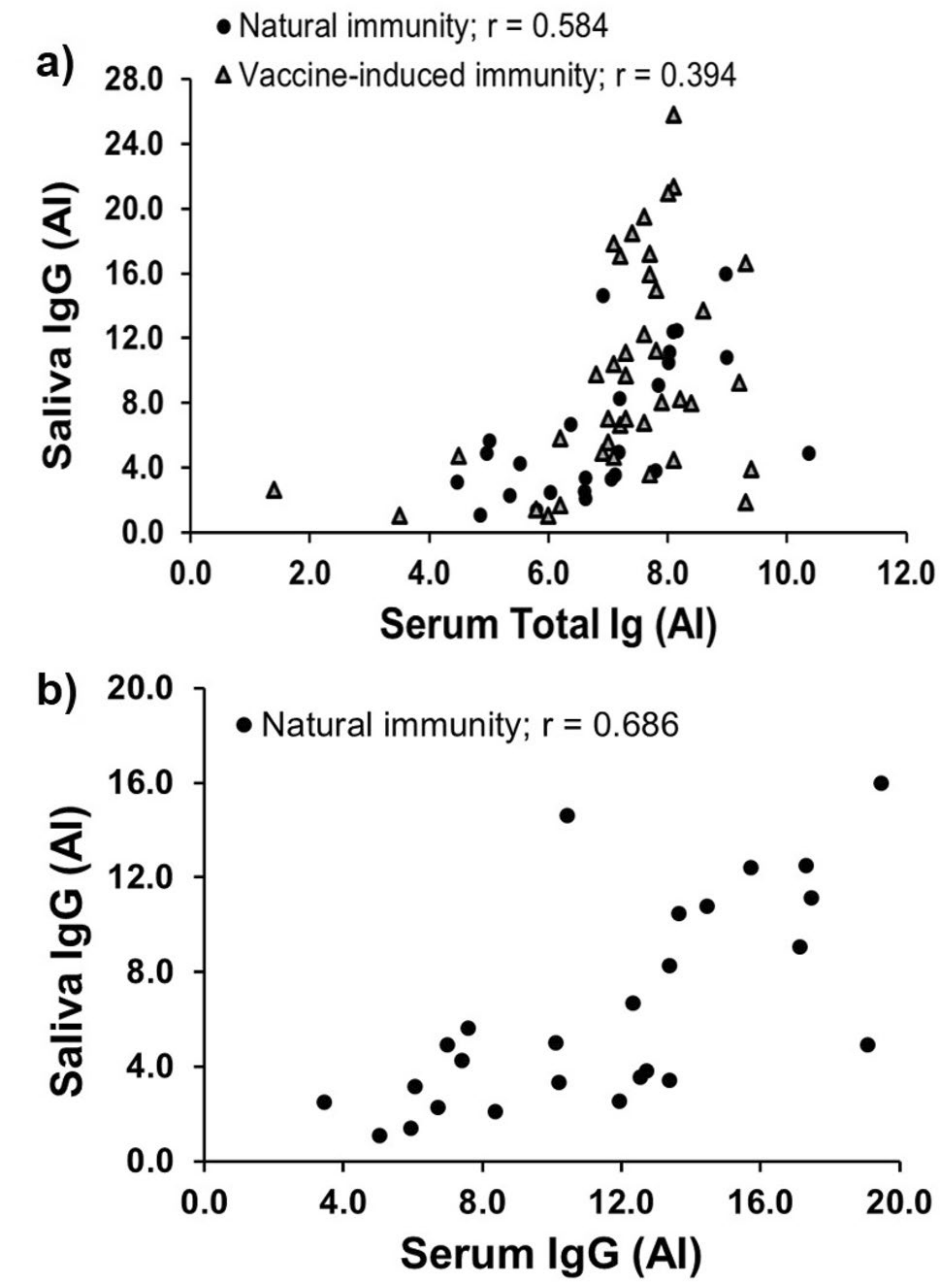
Concordance of SARS-CoV-2 spike protein RBD AI values among serum and saliva samples from individuals with natural and vaccine-induced COVID-19 immunity using the home-brew ELISA. SARS-CoV-2 RBD total antibody levels were measured in serum and SARS-CoV-2 RBD IgG antibody levels were measured in concentrated saliva samples using a laboratory-developed ELISA. (**a**) Total RBD antibody levels in serum and RBD IgG antibody levels in saliva samples from convalescent plasma donors (n = 26) with natural COVID-19 immunity and individuals with vaccine-induced COVID-19 immunity (n = 40). (**b**) RBD IgG antibody levels in saliva and serum from convalescent plasma donors (n = 26) with natural COVID-19 immunity. *AI* antibody index.

Next, we evaluated the concordance of SARS-CoV-2 spike protein RBD AI values among serum and saliva samples from individuals with natural and vaccine-induced COVID-19 immunity (n = 26, and n = 40, respectively). SARS-CoV-2 RBD total antibody and IgG levels were measured in serum and concentrated saliva samples using the laboratory-developed ELISA mentioned above. The correlation between total RBD antibody levels in serum and IgG levels in saliva was 48% higher among the individuals with natural immunity (r = 0.584) compared to the individuals with vaccine-induced COVID-19 immunity (r = 0.394) (Fig. 2a). However, when restricting the analysis to the RBD IgG antibody levels, the correlation between the serum and saliva AI values increased to 0.686 among the individuals with natural COVID-19 immunity (Fig. 2b). Salivary RBD IgG antibody levels among the individuals with vaccine-induced COVID-19 immunity were not assayed.

### Ultrasensitive saliva-based SARS-CoV-2 spike RBD IgG and IgA antibody immunoassay

**Figure 3:**
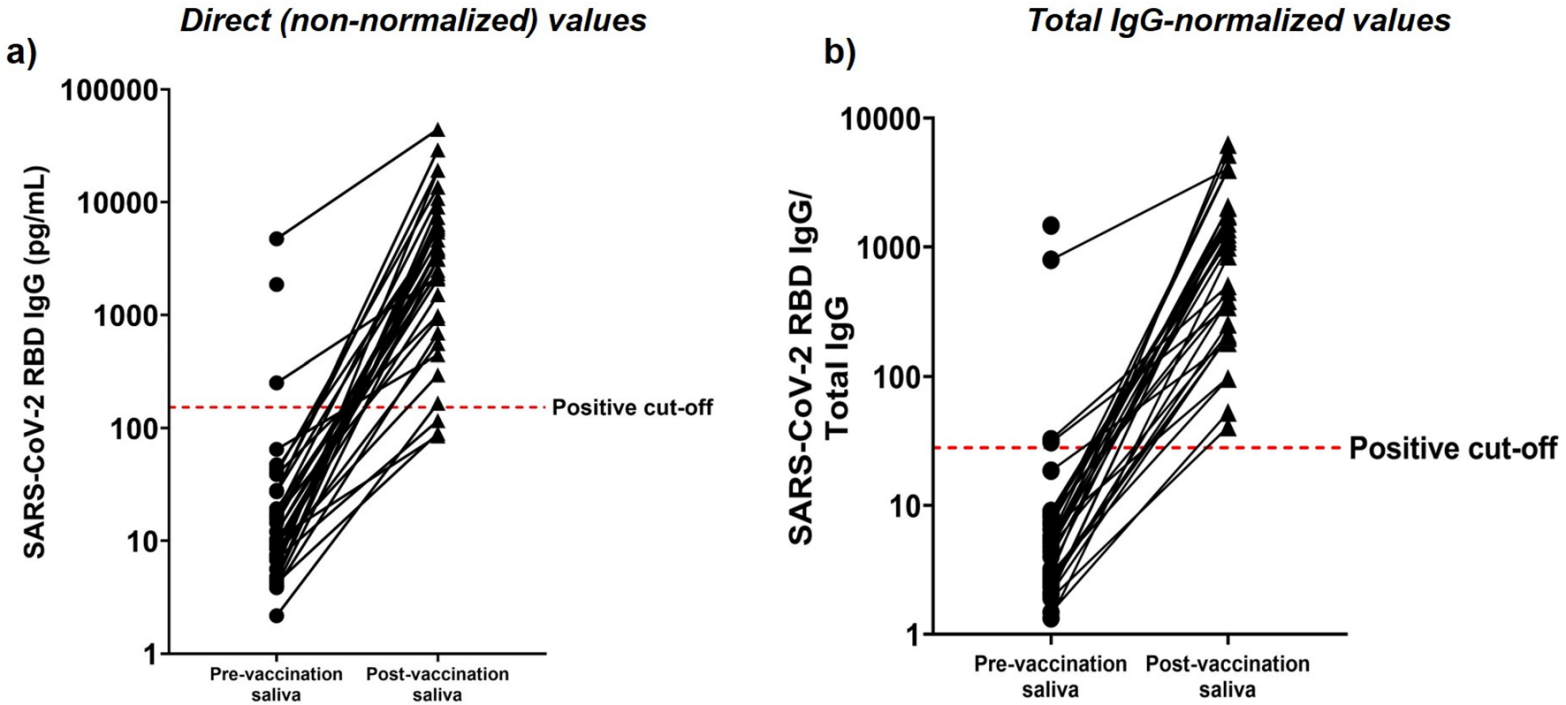
Distribution of SARS-CoV-2 spike protein RBD IgG antibody levels in pre- and post-COVID-19 vaccination unconcentrated saliva samples measured using a Simoa immunoassay with and without total IgG normalization. The levels of SARS-CoV-2 RBD IgG were measured in the saliva from 36 pre-vaccinated individuals and 34 post-vaccinated individuals. The positive cut-off was 153 pg/mL. (**a**) Direct (non-normalized) values. (**b**) RBD IgG values (in ng/mL) normalized to total IgG levels (in ng/mL) and multiplied by 1000.

The analytical sensitivity of the saliva-based Simoa immunoassay was five orders of magnitude higher than the laboratory-developed ELISA assay: 0.24 pg/mL using unconcentrated saliva measured by the Simoa immunoassay, compared to 15 ng/mL using ~ eightfold concentrated saliva measured by the laboratory-developed ELISA. A subset of the same pre-vaccination and post-vaccination paired serum and saliva specimens that were used to evaluate the performance of our laboratory-developed ELISA were used to characterize the performance of the Simoa immunoassay. Based on the distribution of antibody concentrations in the pre-vaccination samples, 153 pg/mL was selected as the positivity cut-off (Fig. 3a). An average relative increase in antibody concentration of 431 pg/mL was observed in the post- vs. pre-vaccination saliva samples. The overall diagnostic sensitivity of the Simoa immunoassay was 91.7% (95% CI 77.5–98.2%) and the specificity was 97.1% (95% CI 84.7–99.9%) (Table 1). Two of the four post-vaccination saliva samples that were negative using the saliva laboratory-developed ELISA method were positive when analyzed using the Simoa immunoassay, which further confirms the enhanced analytical sensitivity of the Simoa immunoassay compared to the ELISA method.

Prior studies have demonstrated that measurement of total salivary IgG levels is important not only to ensure adequacy of immunoglobulins present in the salivary sample for valid serological studies, but also to normalize pathogen-specific antibodies to allow for accurate quantitative comparison of samples^26–29^. Therefore, total IgG levels were measured in each saliva sample and used to normalize the levels of SARS-CoV-2 spike RBD IgG (Fig. 3b). The positivity cutoff was set at 28. Total IgG-based normalization resulted in the re-classification of one true negative result among the pre-vaccination samples as a false positive result and all three of the false negative results among the post-vaccination samples as true positive results (Table 1). Overall, the Simoa immunoassay showed an improvement in sensitivity (from 91.7% to 100%) when assessing total IgG-normalized salivary SARS-CoV-2 spike RBD IgG levels compared to non-normalized levels. However, there was a small decrease in specificity (from 97.1% to 94.1%) with total IgG normalization.

Given that IgG in saliva originates mostly from plasma by transudation from the gingival blood circulation^30–32^ and we were interested in salivary immunoglobulin levels that are correlates of mucosal immunity, we decided to investigate salivary levels of spike RBD IgA using a Simoa immunoassay. Based on the distribution of antibody concentrations in the pre-vaccination samples, 147 pg/mL was selected as the positivity cut-off. The overall diagnostic sensitivity of the IgA assay [41.7% (95% CI 25.5–59.2%)] using unconcentrated saliva was considerably poorer than the IgG assay (Table 1). However, the overall diagnostic specificity of the IgA assay [97.0% (CI: 84.2–99.9%)] approached that of the IgG assay [100% (90.0–100%)]. The positive predictive value and negative predictive values of this assay as well as the IgG Home-brew ELISA, IgG Simoa assay without IgG normalization and IgG Simoa assay with IgG normalization are included in Supplemental Table 1.

The median salivary spike RBD IgA level in the pre-vaccination cohort was four-fold higher compared to the median IgG level, but the median spike RBD IgA level in the post-vaccination cohort was 0.03-fold lower than the median IgG level. Relatedly, there was a greater vaccine-induced median magnitude of increase in the salivary IgG level (261-fold) vs. the IgA level (2.2-fold).

## Discussion

We conducted a detailed validation of a high-sensitivity immunoassay using ultrasensitive Simoa-based technology to accurately measure the lower level of antibodies in saliva relative to serum among individuals after COVID-19 vaccination. Our data support the use of the RBD as an optimal antigenic target for salivary anti-SARS-CoV-2 IgG antibody assays. Additionally, whereas salivary IgA is poorly correlated with serum IgG SARS-CoV-2 antibody levels, the presence of anti-SARS-CoV-2 RBD IgG antibodies in the saliva of individuals with natural and vaccine-induced COVID-19 immunity demonstrates excellent concordance with paired serum samples in our study.

Only a handful of prior studies have assessed the presence of salivary anti-SARS-CoV-2 antibodies in naturally infected^15,17,18,33,34^ or vaccinated individuals^35,36^, but have presented differing results, and have universally acknowledged challenges with measurement of salivary antibodies and a need for improvement of salivary assays. Faustini et al. detected anti-spike SARS-CoV-2 antibodies in the serum of 95% of previously infected patients, but salivary antibodies were detected in only 17.5% of this cohort, primarily in those with higher serum antibody levels^18^. They concluded that their assay may not have been sensitive enough to adequately detect salivary antibodies. Isho et al*.* were the first to report salivary antibody data by isotype in previously infected individuals, reporting significantly higher rates of salivary antibody detection than Faustini et al*.*, with IgG spike and RBD antibody assay sensitivities of 89 and 85%, respectively, in saliva, and lower sensitivities for IgA detection (51% for spike, 30% for RBD)^15^. Pisanic et al*.* developed a multiplex assay employing 12 SARS-CoV-2 antigen targets, with assay sensitivities varying from 46 -100% for salivary IgG and from 14–59% for salivary IgA, depending on the antigenic target^17^. These data from Pisanic et al. indicate that the antigenic target used in salivary antibody assays can greatly influence assay performance characteristics and may partially explain discrepancies in assay performance amongst the currently published studies.

Our study provides several important additions to the current literature on anti-SARS-CoV-2 salivary antibody detection. First, one of the primary challenges of using saliva for antibody detection is the low quantities of antibodies in saliva, relative to serum. Previous studies have demonstrated that the concentrations of antibodies, particularly IgG, in oral specimens are 800- to 1000-fold lower than that in serum^13^. Our data confirms these prior studies, as we found that salivary antibodies were three orders of magnitude lower than serum antibodies; this required concentration of salivary samples to allow detection of salivary antibodies using our laboratory-developed SARS-CoV-2 RBD total antibody ELISA. To address this challenge, we are the first to perform a detailed validation of a high-sensitivity assay using Simoa-based technology to accurately measure low antibody levels in saliva among individuals after COVID-19 vaccination. We were able to demonstrate improvement in diagnostic assay performance characteristics with an ultrasensitive Simoa immunoassay, when compared to our laboratory-developed ELISA. Other studies have described the use of Simoa for the ultrasensitive measurement of SARS-CoV-2 antibodies in saliva^37,38^; however, clinical validation data was not included in these studies. A Simoa semi-quantitative antibody test with Emergency Use Authorization from the US FDA is available for the measurement of SARS-CoV-2 IgG levels in serum and EDTA plasma (https://www.fda.gov/media/144764/download); however, a similar test does not exist for use with saliva.

Second, our study examined the impact of total IgG levels on assay performance characteristics for the ultrasensitive Simoa immunoassay. Preanalytical variability in the saliva collection process can influence the total level of antibodies collected by each study participant. Inadequate collection of salivary immunoglobulins has been shown to impact the accuracy and validity of salivary serological studies. Accordingly, measuring total IgG in oral fluid and normalizing pathogen-specific antibody levels based on the total IgG levels has been demonstrated to be an effective quality control method for saliva-based serological studies^27–29^. Using this method of normalization in our study, one true negative SARS-CoV-2 spike RBD IgG result (65 pg/mL) was re-categorized as a false positive result in the pre-vaccination cohort. Additionally, three false negative results (85, 90, and 117 pg/mL) in the post-vaccination cohort were re-categorized as true positive results following total IgG-based normalization; hence, it is possible that the total antibody levels in these samples were insufficient to enable valid serological measurements. Although total IgG normalization has been shown to significantly improve the performance characteristics of salivary antibody assays in previous studies^27–29^, total IgG normalization for the ultrasensitive Simoa immunoassay used in our study had only a small impact on assay performance, with assay sensitivity modestly increasing from 91.7 to 100% with total IgG normalization, and assay specificity slightly decreasing from 97.1 to 94.1% with total IgG normalization. This minimal impact on assay performance may be due to the high sensitivity of the Simoa assay, as prior studies relying on total IgG normalization utilized less sensitive antibody detection methods.

Third, our data support the use of the RBD as an optimal antigenic target for salivary anti-SARS-CoV-2 IgG antibody assays. Our laboratory-developed ELISA demonstrated high sensitivity (90%) and specificity (92.5%), and the Simoa-based immunoassay had even better performance characteristics (91.7% sensitivity and 97.1% specificity without total IgG normalization; 100% sensitivity, 94.1% specificity with total IgG normalization). These data are consistent with Pisanic et al., who demonstrated high sensitivities and specificities of 88–100% and 98–100%, respectively, for four different RBD antigen targets^17^.

Other salivary antibody studies have demonstrated a poor correlation between serum anti-SARS-CoV-2 antibody levels and salivary anti-SARS-CoV-2 IgA antibodies. Both Isho et al. and Pisanic et al. demonstrated poorer correlations between serum and saliva for IgA, consistent with our data demonstrating a sensitivity of 41.7% for our Simoa salivary IgA assay^15,17^. Our reported sensitivity was nearly identical to the overall sensitivity of Pisanic et al*.*’s multiplex Luminex assay, which had a reported diagnostic sensitivity of 45%. Interestingly, both Isho et al*.* and Pisanic et al*.* reported a high background level of IgA detected in pre-pandemic or COVID-negative saliva. Our median salivary spike RBD IgA level in the pre-vaccination cohort was fourfold higher compared to the median IgG level, confirming an unexplained high level of background with IgA detection. Given that we are now the third study that have described this phenomenon, with three different antibody detection methods, it is reasonable to hypothesize that this represents true cross-reactive IgA antibodies present in saliva that originated from prior viral exposures.

The parotid, submandibular, and minor salivary glands are reservoirs for SARS-CoV-2, and these glands are implicated in the secretion of secretory IgA (SIgA)^39^. SARS-CoV-2 cross-reactive SIgA has been observed in the saliva of individuals who had not previously been infected with SARS-CoV-2, which suggests that SIgA helps prevent SARS-CoV-2 infection^40,41^. This is in agreement with the well-known role of SIgA in preventing infections through mucosal immunity by preventing the entry of antigens from the mucosa^42^.

Limitations of our study include the relatively low number of study participants and the lack of power to identify potential differences in vaccine-specific antibody levels given that the study participants received either the Pfizer/BioNTech BNT162b2 or the Moderna mRNA-1273 vaccine. An additional limitation is the use of a single antigenic target in our laboratory-developed ELISA and Simoa immunoassay. Ongoing efforts by our group are focused on developing a multiplexed Simoa immunoassay to detect anti-SARS-CoV-2 RBD IgG and IgA antibodies along with nucleocapsid IgG and IgA antibodies. These multiplexed Simoa immunoassays could be used to investigate the duration of antibody responses following COVID-19 infection and/or vaccination. It would also be of interest to determine whether salivary antibody levels are associated with SARS-CoV-2 neutralizing activity, as has been demonstrated in serum^19–21^.

In summary, our study confirms that saliva-based SARS-CoV-2 antibody testing has sufficient sensitivity and specificity to be used as a surrogate for serum-based SARS-CoV-2 antibody testing. We present the first reported development of an ultrasensitive salivary antibody detection method which improves upon the assay performance characteristics of our more traditional laboratory-developed ELISA method. We also show that normalization of saliva samples with an ultrasensitive detection method for total immunoglobulins has a mixed impact on assay performance, with improvement in sensitivity of SARS-CoV-2 antibody detection but decreased specificity, and thus the necessity for performing normalization should be evaluated when validating an ultrasensitive method. Lastly, we demonstrate that salivary IgA is poorly correlated with serum antibody levels, which may be partly due to the presence of cross-reactive IgA generated from non-SARS-CoV-2 exposures.

## Supplementary Information

Supplementary Information.

## Abbreviations

AI: Antibody index
COVID-19: Coronavirus disease 2019
ELISA: Enzyme-linked immunosorbent assay
OD: Optical density
RBD: Receptor binding domain
SARS-CoV-2: Severe acute respiratory syndrome coronavirus 2
Simoa: Single-molecule array
