# Supplementary material for: Ultrasensitive detection of salivary SARS-CoV-2 IgG antibodies in individuals with natural and COVID-19 vaccine-induced immunity
Source: Sci Rep. 2022 May 25;12:8890. doi: 10.1038/s41598-022-12869-z (PMC9132168; doi:10.1038/s41598-022-12869-z)
Supplement: Supplementary file 1 — Supplementary Information. [file 41598_2022_12869_MOESM1_ESM.docx]

**Ultrasensitive detection of salivary SARS-CoV-2 IgG antibodies in individuals with natural and COVID-19 vaccine-induced immunity**

Stefani N. Thomas^1§^; Amy B. Karger^1§^; Ghaith Altawallbeh^1§◊^; Kathryn M. Nelson^2^; David R. Jacobs Jr.^3^; Jed Gorlin^4^, Helene Barcelo, Bharat Thyagarajan^1*^

^1^ Department of Laboratory Medicine and Pathology, School of Medicine, University of Minnesota, Minneapolis, MN

^2^ Institute for Therapeutics Discovery and Development, College of Pharmacy, University of Minnesota, Minneapolis, MN

^3^ Division of Epidemiology and Community Health, School of Public Health, University of Minnesota, Minneapolis, MN

^4^ Memorial Blood Centers – a division of New York Blood Center Enterprises, St. Paul, MN

^§^ These authors contributed equally

**Supplemental Table 1. Positive predictive value (PPV) and negative predictive value (NPV) diagnostic performance characteristics of the saliva-based IgG home-brew ELISA, IgG Simoa assay without IgG normalization, IgG Simoa assay with IgG normalization and IgA Simoa assay.**

|  | Pre-vaccination | | Post-vaccination | | Overall | |
| --- | --- | --- | --- | --- | --- | --- |
|  | **PPV** | **NPV** | **PPV** | **NPV** | **PPV** | **NPV** |
| IgG Home-brew ELISA | 40%  [18.4 – 66.4%]  (2/2+3) | 100%  [NA]  (35/35+0) | 100%  [NA]  (34/34+0) | 33.3%  [16.5 – 55.8%]  (2/2+4) | 92.3%  [80.1 – 97.3%]  (36/36+3) | 90.2%  [78.4 – 95.9%]  (37/37+4) |
| IgG Simoa assay  (without IgG normalization) | 66.7%  [22.5 –93.2%]  (2/2+1) | 100%  [NA]  (33/33+0) | 100%  [NA]  (31/31+0) | NA  (0/0+3) | 97.1%  [82.7 – 99.6%]  (33/33+1) | 91.7%  [78.8 – 97.0%]  (33/33+3) |
| IgG Simoa assay  (with IgG normalization) | 50%  [20.7 – 79.3%]  (2/2+2) | 100%  [NA]  (32/32+0) | 100%  [NA]  (34/34+0) | NA  (0/0+0) | 94.7%  [82.4 – 98.6%]  (36/36+2) | 100%  [NA]  (32/32+0) |
| IgA Simoa assay | 50.0%  [8.5 – 91.5%]  (1/1+1) | 97.0%  [88.9 – 99.2%]  (32/32+1) | 100%  [89.7 – 100.0%]  (14/14+0) | 0%  [NA]  (0/0+20) | 93.7%  [67.7 – 99.1%]  (15/15+1) | 60.4%  [53.5 – 66.9%]  (32/32+21) |

Positive predictive value = (True positives/True positives + False positives). Negative predictive value = (True negatives/True negatives + False negatives). [95% confidence interval].

*IgG Home-brew assay:* Two individuals had PCR-confirmed COVID-19 infection prior to vaccination. Both of these saliva samples tested positive in serum and saliva. All other participants (n=38) were confirmed to be COVID-19 negative based on PCR testing. Antibody-positive saliva samples from individuals with antibody-negative serum were considered to be false-positive results. Saliva samples were concentrated prior to analysis.

*IgG Simoa assay without IgG normalization:* The levels of SARS-CoV-2 RBD IgG were measured in paired serum and saliva samples from 36 pre-vaccinated individuals, serum from 35 post-vaccinated individuals, and saliva from 34 post-vaccinated individuals. Saliva samples were not concentrated prior to analysis.

*IgG Simoa assay with IgG normalization:* The levels of SARS-CoV-2 RBD IgG were measured in paired serum and saliva samples from 36 pre-vaccinated individuals, serum from 35 post-vaccinated individuals, and saliva from 34 post-vaccinated individuals (paired serum and saliva samples were available from 33 individuals). Saliva samples were not concentrated prior to analysis. Saliva SARS-CoV-2 spike RBD protein IgG levels were normalized to total IgG levels.

*IgA Simoa assay:* The levels of SARS-CoV-2 RBD IgA were measured in paired serum and saliva samples from 35 pre-vaccinated individuals, and 34 post-vaccinated individuals. Saliva samples were not concentrated prior to analysis.
